# Association Study of N-Methyl-D-Aspartate Receptor Subunit 2B (*GRIN2B*) Polymorphisms and Schizophrenia Symptoms in the Han Chinese Population

**DOI:** 10.1371/journal.pone.0125925

**Published:** 2015-05-28

**Authors:** Yongfeng Yang, Wenqiang Li, Hongxing Zhang, Ge Yang, Xiujuan Wang, Minli Ding, Tianzi Jiang, Luxian Lv

**Affiliations:** 1 Key Laboratory for NeuroInformation of Ministry of Education, School of Life Science and Technology, University of Electronic Science and Technology of China, Chengdu, China; 2 Department of Psychiatry, Henan Mental Hospital, The Second Affiliated Hospital of Xinxiang Medical University, Xinxiang, China; 3 Henan Key Lab of Biological Psychiatry, Xinxiang Medical University, Xinxiang, China; Kunming Institute of Zoology, Chinese Academy of Sciences, CHINA

## Abstract

Schizophrenia (SZ) is a common and complex psychiatric disorder that has a significant genetic component. The glutamatergic system is the major excitatory neurotransmitter system in the central nervous system, and is mediated by N-methyl-D-aspartate (NMDA) receptors. Disturbances in this system have been hypothesized to play a major role in SZ pathogenesis. Several studies have revealed that the NMDA receptor subunit 2B (*GRIN2B*) potentially associates with SZ and its psychiatric symptoms. In this study, we performed a case–control study to identify polymorphisms of the *GRIN2B* gene that may confer susceptibility to SZ in the Han Chinese population. Thirty-four single nucleotide polymorphisms (SNPs) were genotyped in 528 paranoid SZ patients and 528 control subjects. A significant association was observed in allele and genotype between SZ and controls at rs2098469 (*χ^2^* = 8.425 and 4.994; *p* = 0.025 and 0.014, respectively). Significant associations were found in the allele at rs12319804 (*χ^2^* = 4.436; *p* = 0.035), as well as in the genotype at rs12820037 and rs7298664 between SZ and controls (*χ^2^* = 11.162 and 38.204; *p* = 0.003 and 4.27×10^-8^, respectively). After applying the Bonferroni correction, rs7298664 still had significant genotype associations with SZ (*p* = 1.71×10^-7^). In addition, rs2098469 genotype and allele frequencies, and 12820037 allele frequencies were nominally associated with SZ. Three haplotypes, CGA (rs10845849—rs12319804—rs10845851), CC (rs12582848—rs7952915), and AAGAC (rs2041986—rs11055665—rs7314376—rs7297101—rs2098469), had significant differences between SZ and controls (*χ^2^* = 4.324, 4.582, and 4.492; *p* = 0.037, 0.032, and 0.034, respectively). In addition, three SNPs, rs2098469, rs12820037, and rs7298664, were significantly associated with cognition factors PANSS subscores in SZ (*F* = 16.799, 7.112, and 13.357; *p* = 0.000, 0.017, and 0.000, respectively). In conclusion, our study provides novel evidence for an association between *GRIN2B* polymorphisms and SZ susceptibility and symptoms in the Han Chinese population.

## Introduction

Schizophrenia (SZ) is a common, chronic, and complex psychiatric disorder that includes delusions and hallucinations, reduced interest and drive, altered emotional reactivity, and disorganized behavior [1]. SZ affects 1.0% of the worldwide population [2]. Family studies, including twin and adoption studies, provide evidence that SZ is predominantly a genetic disorder, and heritability estimates for SZ range from 60% to 80% [3‒5]. Traditionally, SZ genetic research focused on identifying linkage regions, candidate genes, and polymorphisms. Data indicated susceptibility genes contributed to SZ [6‒9]. Some results suggest that multiple, individual mutations that alter genes in neurotransmitter pathways contribute to SZ [10‒12].

Glutamate (Glu) is the primary excitatory neurotransmitter involved in various neural processes, including neuronal development, synaptic plasticity, and neuronal toxicity in the brain system [13]. In the late 1980s, the Glu hypofunction model of SZ was first suggested, and was based upon the observation that phencyclidine, ketamine, and similarly-acting psychotomimetic compounds induced their unique behavioral effects by blocking neurotransmission at N-methyl-D-aspartate-type glutamate receptors (NMDAR) [10]. Moreover, the glutamate system consists of interconnecting pathways of the cerebral cortex and limbic system, which are brain regions implicated in SZ pathophysiology [11]. As glutamate receptors, NMDARs are ligand- and voltage-gated ion channels that play crucial roles in excitatory synaptic transmission, plasticity, and excitoxicity. They are composed of an N-methyl-D-aspartate-type glutamate (NMDA) receptor NR1 subunit (GRIN1) and one of the four NMDA receptor NR2 subunits (GRIN2A-D) [14‒16]. Among the proposed mechanisms, disturbances in NMDAR-mediated neuronal transmission offer a logical hypothesis for SZ development.

The NR2B subunit is encoded by the NMDA receptor 2B subunit gene (*GRIN2B*; MIN_138252), which is located at chromosome 12p12 and consists of 13 exons. Moreover, variations in *GRIN2B* have been linked to SZ [17,18]. The NMDA subunit NR2B, encoded by *GRIN2B*, has been implicated in cases of mental retardation [19]. Many recent studies have focused on insertion/deletion polymorphisms and explored potential associations between *GRIN2B* and SZ. Excessive rare missense *GRIN2B* mutations have also been reported in people with both SZ and autism [20]. However, other studies had inconsistent results [18,20 **‒** 24]. SZ is highly heterogeneous in both clinical expression and genotype, and it is believed that variations in genotype are related to different clinical subtypes. In the present study, we performed a case–control analysis to investigate the potential association between *GRIN2B* SNPs and SZ symptoms in the Han Chinese population.

## Materials and Methods

### Subjects

The Ethical Committee of the Second Affiliated Hospital of Xinxiang Medical University (China) approved the study protocol. Written informed consent was obtained from all participants after the objectives and procedures of the study were fully explained.

The patient population was recruited from inpatients at the Second Affiliated Hospital of Xinxiang Medical University from March 2005 to December 2008. The population consisted of 528 SZ patients (264 males and 264 females; mean age: 27.32 ± 8.03 years old) and 528 healthy controls (264 males and 264 females; mean age: 27.73 ± 8.01 years old). Patients were unrelated Han Chinese born and living in the North Henan province, and all of their biological grandparents were of Han Chinese ancestry. Consensus diagnoses were made by at least two experienced psychiatrists according to the Diagnostic and Statistical Manual of Mental Disorders-Fourth Edition IV (DSM-IV) (1994) diagnostic criteria for SZ. Only paranoid SZ patients were selected. Individuals with a history of severe medical complications, organic brain disease, other major psychiatric disorders, or substance dependence were excluded.

The age of SZ onset was the age at first manifestation of positive symptoms [25], and was derived from the Comprehensive Assessment of Symptoms and History (CASH) [26]. A “family mental health history” was defined as at least one first- or second-degree relative of the proband who met the DSM-IV criteria for SZ or schizoaffective disorder, and was obtained from the patient or a family member during the initial interview. The raters involved in this study were trained psychiatrists experienced in the administration of psychopathological tests such as the CASH and Positive and Negative Symptom Scale (PANSS). To ensure inter-rater consistency, all participating psychiatrists underwent training every 6 months wherein diagnoses and test results were compared using videotaped demonstration interviews. Agreement among the raters was high for the DSM-IV, CASH, and PANSS, with kappa values ranging from 0.762 to 0.843 and intraclass correlation coefficients (r values) ranging from 0.827 to 0.933.

Two hundred and twenty-nine patients (116 males and 113 females, mean age 27.53 ± 6.01 years, part of the total sample of 528 patients) were evaluated for psychotic syndromes using the PANSS [27] when not taking any antipsychotic medications. Raters were trained to administer the PANSS using the Structured Clinical Interview for the PANSS and achieved an inter-rater reliability of 0.80 or greater. Five factors were derived from PANSS [28]: positive (items P1, P3, P6, G6), negative (N1, N2, N3, N4, N6, G7, G13, G16, P5), expression/anxiety (G1, G2, G3, G4, G6, G12), cognition (P2, N5, N7, G5, G10, G11, G15), and excitement/hostility (P4, P7, G8, G14).

Healthy controls were recruited volunteers from communities and colleges within the same region and matched to the patient group for gender ratio (1:1 for both groups), age (F = 0.699, *p* = 0.403), and Han ethnicity, and with simple non-structured interviews performed by psychiatrists. Any individual with a personal or family history of mental or neurological diseases was excluded. All participants were unrelated Han Chinese who were born and living in the North Henan province. Their biological grandparents were of Han Chinese ancestry.

Peripheral blood samples from each subject were collected into vacutainer tubes containing the anticoagulant ethylene-diaminetetracetic acid. Genomic DNA was extracted from leukocytes using the RelaxGene Blood DNA System (Tiangen Biotech, Beijing, China).

### SNP selection

In this study, *GRIN2B* SNPs were selected for study according to the following criteria: (1) All SNPs covering the genomic region chr12: 13605448−14022988 were included in functional analyses using the FASTSNP online service (http://fastsnp.ibms.sinica.edu.tw.) [29]. Only those SNPs with highly ranked risk and a minor allele frequency (MAF) ≥0.05 in the Chinese Beijing population according to the HapMap database were selected; (2) Tag SNPs were chosen based on the aggressive tagging algorithm (Carlson et al., 2004) (*r* ^*2*^ *≥* 0.80, MAF ≥0.05) using genotype data from the HapMap database as implemented in Haploview v4.1 (http://www.broad.mit.edu/mpg/haploview/) [30]; and, (3) All SNPs selected using these two criteria were assessed using Illumina design scores, and all SNPs with scores below 0.6 were excluded.

### Genotyping

Genotyping was performed using Illumina GoldenGate assays on a BeadStation 500G Genotyping System (Illumina, Inc., San Diego, CA, USA). DNA samples (250 ng) were genotyped according to the Illumina protocol. DNA samples from cases and controls were randomly sorted, including 96 duplicated DNA samples for genotyping quality control. Genotype calls were made using the Genotyping module of the BeadStudio 2.0 software (Illumina, Inc.). All genotype data were examined for cluster separation using Illumina quality scores generated by the software. Poorly performing SNPs were excluded, designated by a GenTrain score <0.4 or a cluster separation score <0.6. SNPs were further excluded from controls not in Hardy–Weinberg equilibrium (HWE). As a genotyping quality control, four SNPs were genotyped in duplicate (100 samples) by DNA sequencing.

### Statistical analyses

Genotypes and alleles were compared between patients and controls using the Golden Helix SVS7.2 program with 10,000 random permutations (Golden Helix, Inc. Bozeman, MT, USA; http://www.goldenhelix.com/). HWE was also calculated using this software. The standardized measure of linkage disequilibrium (LD), coefficients (D′), haplotype frequency, haplotype block, and haplotype association was assessed using the Haploview V4.1 program [30].

Single-SNP analyses for individual genotyping data were performed using Pearson’s chi-square tests on allele and genotype counts. Correlations between alleles and SZ were performed using Armitage trend tests. Odds ratio (OR) and 95% confidence intervals (95% CI) were calculated to evaluate the effect of different alleles and haplotypes. HWE was assessed using chi-square tests with one degree of freedom. Haplotype frequencies were estimated using the expectation maximization (EM) algorithm. To evaluate interactions between genes and sex, global test for interaction was performed. *p*<0.05 was considered statistically significant for tests with expected HWE. Power analyses were performed using the Genetic Power Calculator for this study [31]. Genotyping data were analyzed using Structure 2.3 (http://pritchardlab.stanford.edu/structure_software/release_versions/v2.3.4/html/structure.html) to generate population stratification assignments for all individuals using the Markov chain Monte Carlo (MCMC) algorithm [32].

Genotype differences between SZ patients and healthy controls were compared using chi-square tests (1 df). Associations between age-at-onset subgroups and different genotype carriers were tested using one-way analysis of variance (ANOVA) tests (SPSS version 13.0, SPSS, Inc. Chicago, IL, USA). Differences between *GRIN2B* genotypes in the SZ group and scores of five factors from the PANSS were examined using ANOVA with age, age at onset and illness duration as covariables. Fisher’s least significant difference (LSD) tests were used for pair-wise comparisons of the three genotypes following one-way ANOVA tests. The Bonferroni correction for multiple pair-wise comparisons was conducted for the (X × phenotype) interaction to reduce the probability of false positives, as the probability of at least one chance rejection of the null hypothesis (the product of α and the number of possible comparisons) was *p* = 0.25. The corrected α′ (*p* = 0.01) is α (*p* = 0.05) divided by the number of possible comparisons. Bonferroni corrections for multiple tests were performed to exclude type I errors.

## Results

To reveal allelic variants of the *GRIN2B* gene that are associated with SZ, we analyzed the allele and genotype frequencies of thirty-four common SNPs in 528 SZ patients and 528 controls of Han Chinese descent. For power test, we set the parameters as follows: SNP site = rs2098469, high risk allele frequency (A) = 0.16, prevalence = 0.1 (prevalence of SZ), genotype relative risk Aa = 1.12, genotype relative risk AA = 2.96 (1.12 and 2.96 were used the online software to analyzed. http://bioinfo.iconcologia.net/snpstats/start.ht), number of cases = 528. For a power above 80%, 392 cases are needed according to Genetic Power Calculator, so for genotype analyses, the sample size (n = 528) had sufficient power (0.70–0.80) to detect effects. Power analyses revealed that the total sample size (n = 1056) had a power of 0.86 to detect a small effect (r = 0.1–0.23), and a power of 1.00 to detect both medium (r = 0.24–0.36) and large (r>0.37) effects on genotype distributions. For allele frequency, the sample size (n = 2112) had the power (0.91–1.00) to detect small, medium, and large effects. The sampling success rate for all subjects and SNPs was 99.75%, and the genotype concordance between the BeadStation 500G Genotyping System and DNA sequencing was 99.25%. Evaluation of population structure using 10,000 iterations for the burn-in period and 10,000 repeats after burn-in revealed no evidence of population stratification in the control group (*K* = 1, *p* = 1).

None of the genotype distributions of these thirty-four SNPs significantly deviated from HWM. There was a significant association in allele at rs2098469 and rs12319804 between SZ and controls (*p* = 0.025 and 0.035, respectively, Table 1). Significant associations were found in the genotypes between SZ and controls at rs2098469, rs12820037, and rs7298664 (*p* = 0.014, 0.003, and 4.27×10^-8^, respectively, Table 1). After applying the Bonferroni correction, rs7298664 still had significant genotype associations with SZ (*p* = 1.71×10^-7^). In addition, significant differences were found in the genotypes of males (rs7298664) and females (rs2098469, rs12820037, and rs1806201) between SZ and controls when the two groups were subdivided by gender (*p* = 8.43×10^-18^, 0.036, 9.5×10^-5^, and 0.018, respectively; S1 Table). After applying the Bonferroni correction, rs7298664 and rs12820037 still had significant genotype associations with SZ in male and female respectively (*p* = 3.37×10^-17^ and 3.7×10^-4^, respectively; S1 Table).

**Table 1:** Genotype and allele frequencies of thirty-four SNPs in the *GRIN2B* gene in SZ patients and controls.

SNP#	dbSNP ID	Allele(D/d)^a^	Patients						Controls						*p*-value			*Corrected p*-value	
			n^b^	HWE(*p*)	Genotype			MAF	n^b^	HWE(*p*)	Genotype			MAF					
					DD	Dd	dd				DD	Dd	dd		Genotype	Allele	Genotype		Allele	
1	rs1805502	A/G	527	0.973	337	169	21	0.200	528	0.230	348	156	24	0.193	0.639	0.685	1		1	
2	rs1805476	A/C	524	0.880	321	179	24	0.217	527	0.657	334	169	24	0.206	0.764	0547	1		1	
3	rs890	A/C	528	0.065	286	216	26	0.254	528	0.536	312	191	25	0.228	0.261	0.169	1		1	
4	rs1805247	A/G	527	0.939	339	167	21	0.198	528	0.253	347	157	24	0.194	0.740	0.809	1		1	
5	rs1806191	G/A	527	0.706	510	17	0	0.016	528	0.740	513	15	0	0.014	0.715	0.717	1		1	
6	rs1806201	G/A	526	0.001	114	310	102	0.489	525	0.398	133	272	120	0.488	0.964	0.067	1		1	
7	rs1805522	G/A	527	0.686	347	163	17	0.187	528	0.303	361	147	20	0.177	0.510	0.558	1		1	
8	rs1805482	G/A	527	0.723	511	16	0	0.015	527	0.774	514	13	0	0.012	0.572	0.574	1		1	
9	rs10845849	A/C	527	0.390	300	200	27	0.241	528	0.962	326	178	24	0.214	0281	0.139	1		1	
10	rs12319804	A/G	528	0.811	334	173	21	0.204	527	0.561	363	151	13	0.168	0.101	0.035*	1		1	
11	rs10845851	A/G	528	0438	172	251	105	0.437	528	0.392	168	251	109	0.444	0.940	0725	1		1	
12	rs12582848	C/A	527	0.878	147	261	119	0.473	527	0.589	144	257	126	0.483	0.877	0.662	1		1	
13	rs7952915	C/G	527	0.306	239	224	64	0.334	528	0.998	217	243	68	0.359	0376	0.228	1		1	
14	rs2041986	G/A	528	0.883	242	232	54	0.322	528	0.512	242	226	60	0.328	0.821	0.780	1		1	
15	rs11055665	G/A	527	0.542	220	236	71	0.359	527	0.746	209	243	75	0.373	0.781	0.497	1		1	
16	rs7314376	G/A	528	0.895	322	180	26	0.220	528	0.163	320	189	19	0.215	0.518	0.791	1		1	
17	rs7297101	A/C	527	0.601	165	265	97	0.435	528	0.064	190	236	102	0.417	0.168	0.382	1		1	
18	rs2098469	A/C	528	0.199	372	147	9	0.156	528	0.139	349	154	25	0.193	0.014 *	0.025*	0.448		0.997	
19	rs10459061	C/A	528	0.245	185	244	99	0.419	528	0.179	193	240	95	0.407	0.867	0.595	1		1	
20	rs219876	G/ A	527	0.773	433	90	4	0.093	528	0.164	447	75	6	0.082	0.370	0.389	1		1	
21	rs7295850	C/G	527	0.543	298	200	29	0.245	528	0.770	302	193	33	0.245	0.815	0.979	1		1	
22	rs219905	C/A	528	0.392	144	254	130	0.487	527	0.308	153	251	123	0.472	0.785	0.484	1		1	
23	rs219913	A/G	528	0.086	470	54	4	0.059	528	0.495	466	61	1	0.060	0.325	0.926	1		1	
24	rs1558908	G/A	526	0.689	179	253	96	0.421	527	0.0208	197	229	101	0.409	0.335	0.560	1		1	
25	rs12829455	G/A	527	0.042	465	58	5	0.064	528	0.409	462	65	1	0.063	0.214	0.929	1		1	
26	rs17221245	A/G	525	0.919	309	187	29	0.233	524	0.497	301	196	27	0.239	0.824	0.778	1		1	
27	rs12820037	A/G	526	0.000	416	92	18	0.122	525	0242	421	101	3	0.102	0.003 *	0.150	0.094		1	
28	rs219936	A/G	523	0.223	141	250	137	0.496	528	0.223	138	250	140	0.498	0.968	0.794	1		1	
29	rs11055697	A/G	527	0.950	171	259	97	0.430	526	0.783	173	260	93	0.424	0.952	0.786	1		1	
30	rs12824372	A/G	528	0.832	261	219	48	0.298	525	0.644	265	215	48	0.295	0.966	0.848	1		1	
31	rs7298664	A/G	527	0.532	341	163	23	0.198	528	0.000	385	88	55	0.188	**4.27×10** ^**–8**^ *	0.529	**1.71×10** ^**–7**^ *		1	
32	rs10505778	G/A	528	0.625	166	265	97	0.435	528	0.958	164	260	104	0.443	0.859	0.693	1		1	
33	rs1421108	A/G	524	0.024	175	264	65	0.391	527	0.098	177	260	70	0.394	0.896	0.868	1		1	
34	rs12581502	G/A	528	0.527	252	221	55	0.313	528	0.313	260	214	54	0.305	0.883	0.671	1		1	

* *p*<0.05;

a Major/minor allele, major and minor alleles are denoted by D and d, respectively;

b Number of samples that are well genotyped.

**Fig 1:**
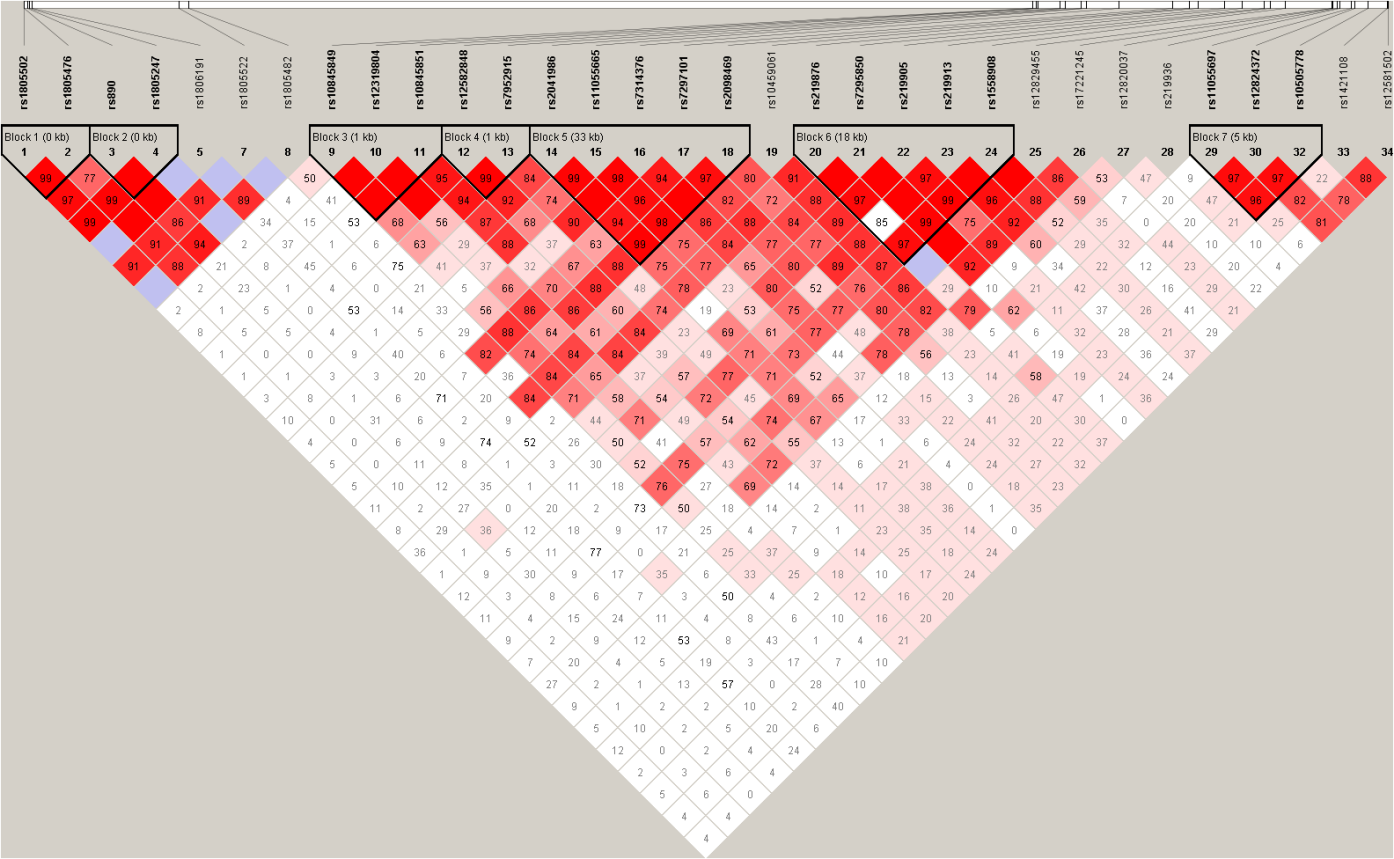
Haplotype block structure of the *GRIN2B* gene in both SZ patients and health controls. Thirty-four SNPs formed seven LD blocks. The index association SNP is represented by a diamond. The color of the remaining SNPs (circles) indicates LD with the index SNP based on pairwise r^2^ values from our data.

To further analyze the haplotype structure in our sample, we evaluated pair-wise LD of thirty-four SNPs in the SZ patient and control group using the standardized D′ and r^2^ values. The position of these SNPs in *GRIN2B*, the LD structure, and the D′ values for all variants are shown in Fig 1. The LD maps for SZ patient and control samples were presented in S1 Fig and S2 Fig respectively. Thirty-four SNPs formed seven LD blocks. Within these blocks, twenty-seven haplotypes were formed, but only three haplotypes, CGA, CC, and AAGA in block 3, 4, and 5, had significantly differed between SZ and controls (*p* = 0.037, 0.032, and 0.034, respectively). Table 2 shows the three significantly associated haplotypes.

**Table 2:** Frequencies of three schizophrenia associated haplotypes in SZ patients and controls.

Block	Haplotype	Haplotype frequencies		χ^2^	*p*-value
		Patients	Controls		
3 (rs10845849- rs12319804-rs10845851)	CGA	0.168	0.204	4.324	0.037
4 (rs12582848-rs7952915)	CC	0.158	0.194	4.582	0.032
5 (rs2041986-rs11055665-rs7314376-rs7297101-rs2098469)	AAGAC	0.189	0.154	4.492	0.034

To investigate the association between *GRIN2B* variations and symptoms, 228 SZ patients with complete PANSS scores were selected. As shown in Table 3, three SNPs positively associated with SZ, and also significantly associated with total PANSS and other five factor scores. Other SNPs from these samples were not found to have these associations. Furthermore, there were significant associations between rs2098469 and total PANSS and five factor subscores with age, age at onset, and illness duration as covariables (*p* = 0.000, S2 Table). Two SNPs, rs12319804 and rs12820037, were significantly associated with positive and cognition factor subscores (*p* = 0.000, 0.000 and *p* = 0.025, 0.003, respectively).

**Table 3:** Association analyses between five factors of PANSS and three *GRIN2B* SNPs in patients with SZ.

SNP	Genotype	Total PANSS	Positive	Negative	Depression/anxiety	Cognition	Excitement/hostility
rs2098469	AA(168)	90.80±22.02^*^	14.80±3.34^*^	25.62±8.36^*^	15.06±5.18^*^	14.64±5.70^*^	12.41±4.80^*^
	AC(57)	95.54±20.77^*^	15.53±3.19^*^	27.04±8.84^*^	15.66±4.75^*^	15.51±5.80^*^	13.16±4.39^*^
	CC(3)	75.33±15.94^*^	—	75.33±15.94^*^	—	—	—
rs12820037	AA(175)	92.68±21.89^**a**^	15.07±3.24	26.43±8.77^**a**^	15.07±5.12^**b**^	14.97±5.85^**b**^	12.73±4.78^**a**^
	AG(44)	88.03±22.57^**c**^	14.60±3.61	23.79±7.67	15.09±5.07^**b**^	14.53±5.56^**b**^	12.13±4.82
	GG(9)	92.90±13.17	14.89±2.80	25.54±6.16^**a**^	17.15±3.58	13.14±2.81	12.35±2.50
rs7298664	AA(144)	91.09±20.57^**b**^	14.85±2.95^**b**^	25.88±8.01	15.34±5.04^*^	14.49±5.41^*^	12.12±4.84^**b**^
	AG(76)	92.36±23.22^**b**^	14.87±3.43^**b**^	26.08±9.55	14.29±4.51^*^	15.20±6.09^*^	13.44±4.27^**c**^
	GG(8)	99.27±27.02	17.82±±5.54	24.93±7.95	19.42±7.15^*^	16.80±6.64^*^	13.43±5.34

* *p*<0.05, compared with each genotype, LSD tests

“—” indicates the genotype was not determined

**a** *p*<0.05, compared with AG genotype, LSD tests

**b** *p*<0.05, compared with GG genotype, LSD tests

**c** *p*<0.05, compared with AA genotype, LSD tests

## Discussion

This study aimed to investigate the *GRIN2B* mutations associated with SZ and psychotic symptoms in the Han Chinese population. Significant differences were observed in genotype frequencies of three SNPs, rs2098469, rs12820037, and rs7298664, between patients and controls. In addition, the allele frequencies of the rs12319804 and rs2098469 SNPs were significantly associated with SZ in this population. Furthermore, the rs7298664 genotype association remained after Bonferroni correction. In addition, the rs2098469 genotype and allele frequencies, and the 12820037 allele frequencies were nominally associated with SZ. Our results found novel variants can influence SZ risk in the Han Chinese population.

Previous studies revealed that dysfunction of the glutamatergic signaling system was a major mechanism in SZ pathogenesis, and genes encoding NMDA receptors are obvious candidates in numerous association studies of SZ [33‒37]. More studies have focused on the association of *GRIN2B* with SZ. The original studies reported that some *GRIN2B* SNPs were significantly associated with SZ [20,22,23,38‒40]. In addition, the rs1019385 SNP was associated with SZ in several studies and meta-analyses [22,34,35,38]. However, other studies found no such associations [18,21,24]. Our study could not validate the positive association of rs1019385; however, novel variants of *GRIN2B*, including rs2098469, rs12820037, and rs7298664 had significant association in genotype frequency with SZ. We also found significant association with SZ when the two groups were subdivided by gender. Moreover, our results are consistent with previous studies that reported no association of rs1805247, rs1806191, rs1806201, rs3026160, rs1805522, rs1805482, and rs35025065 with SZ [18,41]. The present study revealed significant association in alleles between SZ and controls at rs12319804 and rs2098469, which differs from data in previous studies [38,40]. Further association analyses of haplotypes consisting of thirty-four SNPs at the *GRIN2B* gene with SZ were performed. Three haplotypes at *GRIN2B* significantly associated with SZ. In previous studies, better memory performance was observed in the absence of the T-T haplotype at *GRIN2B* rs220599 and rs12828473 [42,43]. Our results were inconsistent with these previous studies [18,22,23]. There are several explanations for these disparate findings. First, the small sample size and high phenotypic heterogeneity likely contributed to low statistical power, such as the conclusions of Martucci *et al*. [22]. Therefore, by including only paranoid SZ patients and enlarging the sample size (528 SZ patients and 528 controls), we potentially improved the power to detect disease associations. In addition, more markers were tested in this study compared to previous reports. Second, differences in population ethnicity and stratification may lead to the high heterogeneity of samples. All of our subjects were living in the North Henan province and belonged to the same population group based on structure analyses. Third, because SZ is a complex genetic disease and genome-wide association studies have revealed multiple susceptibility genes that contribute to SZ pathogenesis [8,9], each gene might exert weak-to-moderate effects.

SZ is characterized by several symptom domains: positive symptoms, negative symptoms, disorganization of thoughts and behaviors, and cognitive deficits [44,45]. Several studies have explored the associated between *GRIN2B* and cognitive deficit symptoms [21,42,43], differential language lateralization [46], anti-psychotic-induced movement disorders [47], and clozapine-induced obsessive-compulsive symptoms [48]. However, there are few studies regarding positive and negative symptoms of SZ and *GRIN2B*. Genetic enhancement of *GRIN2B* may improve learning and memory in mice, and NMDA dysfunction has been related to cognitive impairment associated with SZ [49,50]. Several studies have revealed the association between *GRIN2B* and clinically-manifested cognitive deficit symptoms, including memory performance [42,43]. Currently, neurocognitive dysfunction is a core feature of SZ and recognized in more SZ patients [51]. Therefore, we investigated this feature in our SZ patients using five factors of PANSS. In our study, three SNPs, rs2098469, rs12820037, and rs7298664, were associated with positive, negative, expression/anxiety, and cognition factor PANSS subscores in SZ patients. These differ with former data regarding rs12828473 and rs220599 [42,43]. The differences may be due to unknown population stratification [18], limited sample size [22], incomplete information, or sample heterogeneity [23]. Thus, all results revealed that genetic variations of *GRIN2B* influence symptom traits in SZ. Moreover, rs2098469, rs12820037, and rs7298664 were associated with cognition factor subscores in SZ patients with age, age at onset, and illness duration as covariables. Therefore, our study provided novel evidence for the association of *GRIN2B* with cognition deficit symptoms.

SZ is influenced by glutamatergic neurotransmission and other neurotransmitter networks, including serotonin and dopamine systems. Additionally, we had previously found an association between *SLC6A4* in the serotonin system and SZ [52]. Hence, further studies should examine the interaction of different neurotransmission systems, and how candidate genes affect glutamatergic and related signaling pathways and alter SZ symptoms.

This study had some limitations. First, our sample size may not be large enough for complete PANSS scores. Second, we only selected paranoid SZ patients, not included other subtypes (catatonic, collapse, residual and undifferentiated). Third, this study was limited by a lack of independent validation. Therefore, the further research will need improving in this aspects.

## Conclusion

In summary, our study provides novel data suggesting an association between *GRIN2B* and SZ susceptibility and symptoms. Other studies in different ethnic populations, particularly in patients with defined SZ phenotypes, are required to confirm the role of *GRIN2B* variants in paranoid SZ.

## Supporting Information

S1 Fig Haplotype block structure of the *GRIN2B* gene in SZ patients. Thirty-four SNPs formed seven LD blocks. The index association SNP is represented by a diamond. The color of the remaining SNPs (circles) indicates LD with the index SNP based on pairwise r^2^ values from our data.
(TIF)

S2 Fig Haplotype block structure of the *GRIN2B* gene in health controls. Thirty-four SNPs formed six LD blocks. The index association SNP is represented by a diamond. The color of the remaining SNPs (circles) indicates LD with the index SNP based on pairwise r^2^ values from our data.
(TIF)

S1 Table Genotypic and allelic frequencies of thirty-four SNPs from the chi-square test in female and male samples. (PDF)

S2 Table Association analyses between five factors of PANSS and three *GRIN2B* SNPs with three covariables in patients with SZ. (PDF)
