# Supplementary figures and images for: Association Study of N-Methyl-D-Aspartate Receptor Subunit 2B (GRIN2B) Polymorphisms and Schizophrenia Symptoms in the Han Chinese Population
Source: PLoS One. 2015 May 28;10(5):e0125925. doi: 10.1371/journal.pone.0125925 (PMC4447394; doi:10.1371/journal.pone.0125925)

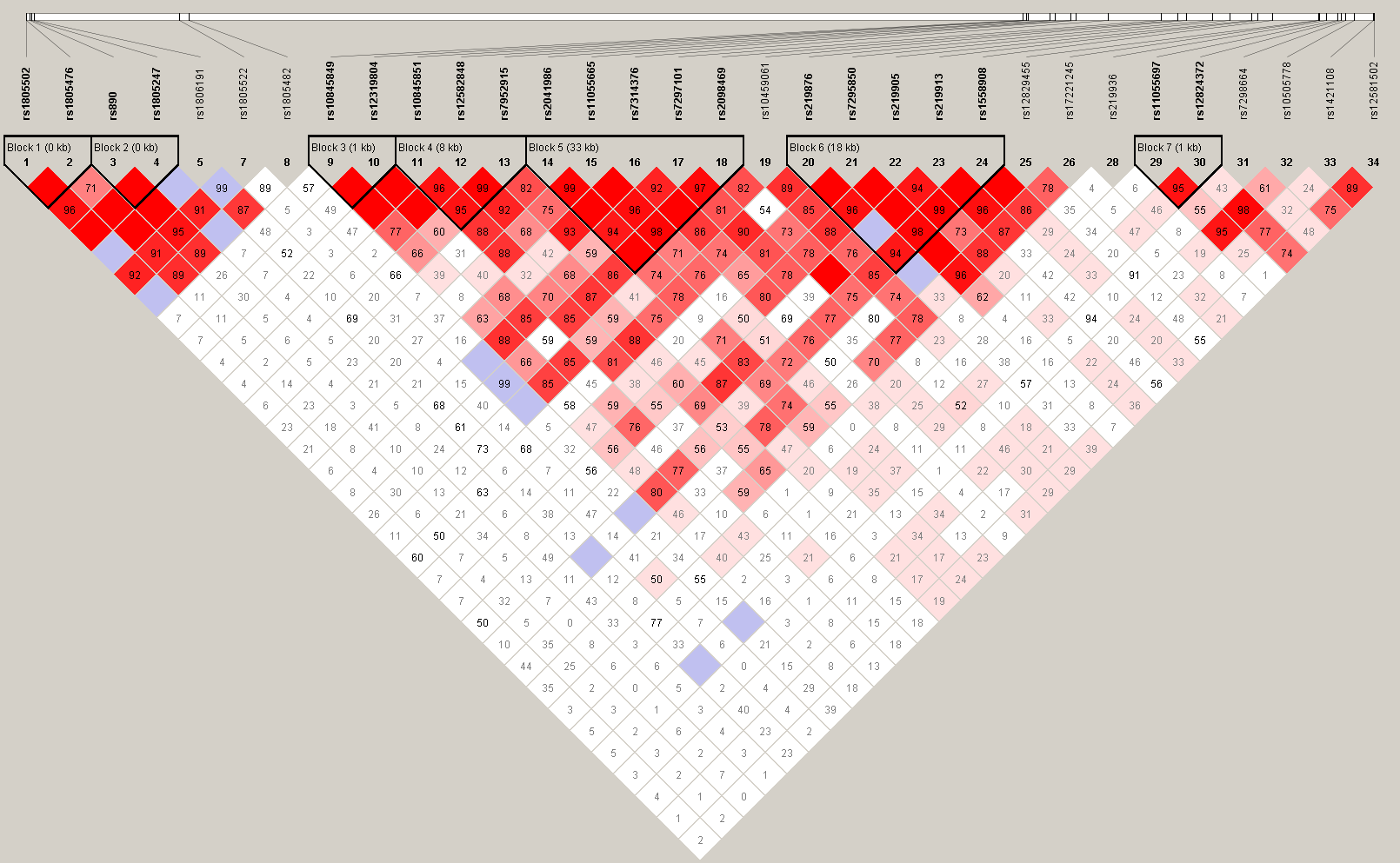

Supplement: S1 Fig — Thirty-four SNPs formed seven LD blocks. The index association SNP is represented by a diamond. The color of the remaining SNPs (circles) indicates LD with the index SNP based on pairwise r2 values from our data. (TIF) [file pone.0125925.s001.tif]

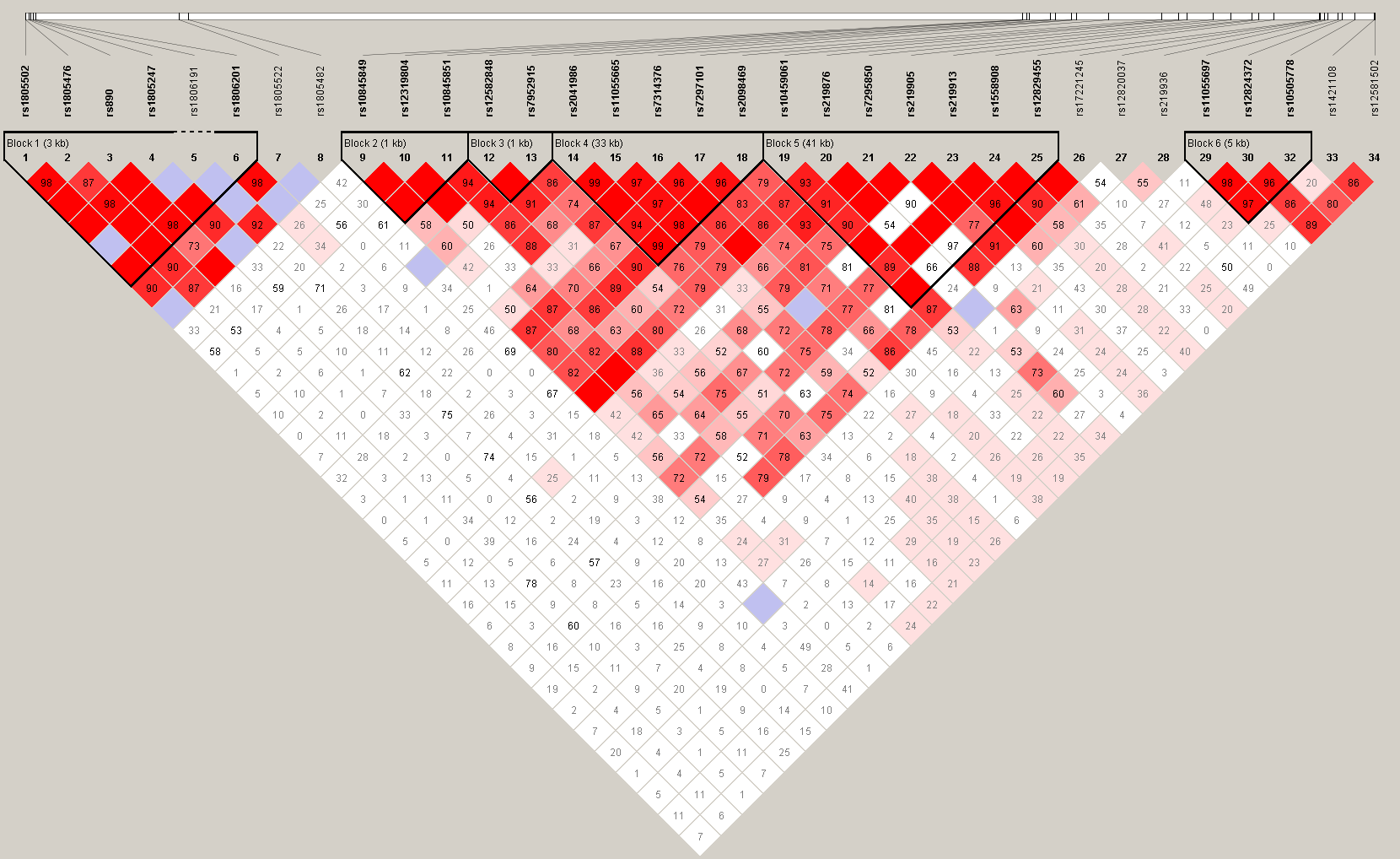

Supplement: S2 Fig — Thirty-four SNPs formed six LD blocks. The index association SNP is represented by a diamond. The color of the remaining SNPs (circles) indicates LD with the index SNP based on pairwise r2 values from our data. (TIF) [file pone.0125925.s002.tif]
