# Supplementary material for: Association Study of N-Methyl-D-Aspartate Receptor Subunit 2B (GRIN2B) Polymorphisms and Schizophrenia Symptoms in the Han Chinese Population
Source: PLoS One. 2015 May 28;10(5):e0125925. doi: 10.1371/journal.pone.0125925 (PMC4447394; doi:10.1371/journal.pone.0125925)
Supplement: S1 Table — (PDF) [file pone.0125925.s003.pdf]

S1 Table. Genotypic and allelic frequencies of thirty-four SNPs from the chi-square test in female and male samples

			Male								Female							
SNP#	dbSNP ID	Allele (D/d)[a]	Patients			Controls			*p*-value		Patients			Controls			*p*-value	
			DD	Dd	dd	DD	Dd	dd	Genotype	Allele	DD	Dd	dd	DD	Dd	dd	Genotype	Allele
1	rs1805502	A/G	160	91	12	17	79	11	0.478	0.271	177	78	9	174	77	13	0.684	0.581
2	rs1805476	A/C	150	96	15	167	86	11	0.357	0.151	171	83	9	167	83	13	0.678	0.537
3	rs890	A/C	143	107	14	157	94	13	0.465	0.280	143	109	12	155	97	12	0.553	0.387
4	rs1805247	A/G	161	90	12	173	80	11	0.588	0.343	178	77	9	174	77	13	0.679	0.528
5	rs1806191	G/A	252	11	0	255	9	0	0.642	0.645	258	6	0	258	6	0	1	1
6	rs1806201	G/A	74	142	46	69	131	62	0.224	0.193	40	168	56	64	141	58	**0.018 (0.072)**	0.175
7	rs1805522	G/A	168	87	8	183	73	8	0.393	0.251	179	76	9	178	74	12	0.795	0.749
8	rs1805482	G/A	251	12	0	255	9	0	0.498	0.502	260	4	0	259	4	0	0.995	0.995
9	rs10845849	A/C	153	97	13	163	87	14	0.639	0.533	147	103	14	163	91	10	0.327	0.143
10	rs12319804	A/G	171	83	10	179	77	8	0.729	0.428	163	90	11	184	74	5	0.078	**0.028 (0.112)**
11	rs10845851	A/G	80	132	52	84	128	52	0.923	0.804	92	119	53	84	123	57	0.750	0.456
12	rs12582848	C/A	65	137	61	71	132	61	0.837	0.712	82	124	58	73	125	65	0.630	0.325
13	rs7952915	C/G	120	114	29	111	122	31	0.709	0.460	119	110	35	106	121	37	0.514	0.334
14	rs2041986	G/A	120	123	21	122	114	28	0.507	0.740	122	109	33	120	112	32	0.964	0.947
15	rs11055665	G/A	108	124	32	105	123	36	0.868	0.654	112	112	39	104	120	39	0.751	0.609

16	rs7314376	G/A	163	91	10	158	95	11	0.899	0.653	159	89	16	162	94	8	0.242	0.412
17	rs7297101	A/C	77	135	51	94	123	47	0.299	0.194	88	130	46	96	113	55	0.310	0.950
18	rs2098469	A/C	189	70	5	173	81	10	0.204	0.086	183	77	4	176	73	15	**0.036 (0.144)**	0.147
19	rs10459061	C/A	86	124	54	89	131	44	0.531	0.418	99	120	45	104	109	51	0.598	0.949
20	rs219876	G/ A	222	39	2	228	34	2	0.810	0.551	211	51	2	219	41	4	0.386	0.535
21	rs7295850	C/G	145	106	12	152	94	18	0.352	0.971	153	94	17	150	99	15	0.867	0.942
22	rs219905	C/A	70	127	67	63	142	59	0.424	0.950	74	127	63	90	109	64	0.229	0.352
23	rs219913	A/G	233	30	1	231	32	1	0.964	0.799	237	24	3	235	29	0	0.175	0.893
24	rs1558908	G/A	87	128	49	86	131	47	0.959	0.950	92	125	47	111	98	54	0.062	0.443
25	rs12829455	G/A	231	32	1	230	33	1	0.991	0.900	234	26	4	232	32	0	0.098	0.799
26	rs17221245	A/G	157	92	15	155	95	11	0.719	0.789	152	95	14	146	101	16	0.806	0.516
27	rs12820037	A/G	207	56	0	220	42	1	0.183	0.207	209	36	18)	201	59	2	**9.5×10^{-5} (3.7×10^{-4})**	0.420
28	rs219936	A/G	66	124	74	68	128	68	0.840	0.622	75	126	63	70	122	72	0.658	0.388
29	rs11055697	A/G	81)	138	44	86	129	48	0.730	0.950	90	121	53	87	131	45	0.577	0.748
30	rs12824372	A/G	128	115	21	140	99	25	0.353	0.587	133	104	27	125	116	23	0.542	0.788
31	rs7298664	A/G	143	97	23	193	16	55	**8.43×10^{-18} (3.37×10^{-17})**	0.216	198	66	0	192	72	0	0.552	0.583
32	rs10505778	G/A	79	144	41	92	119	53	0.086	0.950	87	121	56	72	141	51	0.204	0.536
33	rs1421108	A/G	90	126	33	84	129	34	0.882	0.669	85	138	32	93	131	36	0.694	0.851
34	rs12581502	G/A	134	100	30	131	105	28	0.893	0.946	118	121	25	129	109	26	0.566	0.507

a Major/minor allele, major and minor alleles are denoted by D and d, respectively;
Parentheses is the correcred *p*-value.
