# Supplementary material for: Association Study of N-Methyl-D-Aspartate Receptor Subunit 2B (GRIN2B) Polymorphisms and Schizophrenia Symptoms in the Han Chinese Population
Source: PLoS One. 2015 May 28;10(5):e0125925. doi: 10.1371/journal.pone.0125925 (PMC4447394; doi:10.1371/journal.pone.0125925)
Supplement: S2 Table — (PDF) [file pone.0125925.s004.pdf]

S2 Table. **Association analyses between five factors of PANSS and three *GRIN2B* SNPs with three covariables in patients with SZ**

	rs2098469			rs12820037			rs7298664		
	AA	AC	CC	AA	AG	GG	AA	AG	GG
Age(year)	28.15±8.48	24.40±7.15	34.00±2.98	27.22±8.45	27.60±7.68	26.21±8.18	27.64±8.23	26.16±8.42	29.98±7.28
Age at onset(year)	24.20±7.41	21.08±5.86	33.33±2.52	23.55±7.27	23.34±7.08	23.46±6.87	23.47±6.96	22.99±7.32	28.36±8.52
Illness duration(day)	1472±1777	1264±1321	300±85	1360±1639	1665±1855	1017±964	1577±1868	1166±1220	582±643
Total PANSS	90.80±22.02*	95.54±20.77*	75.33±15.94*	92.68±21.89	88.03±22.57	92.90±13.17	91.09±20.57	92.36±23.22	99.27±27.02
Positive	14.80±3.34*	15.53±3.19*	—	15.07±3.24*	14.60±3.61*	14.89±2.80*	14.85±2.95*	14.87±3.43*	17.82±±5.54*
Negative	25.62±8.36*	27.04±8.84*	75.33±15.94*	26.43±8.77	23.79±7.67	25.54±6.16	25.88±8.01	26.08±9.55	24.93±7.95
Depression/anxiety	15.06±5.18*	15.66±4.75*	—	15.07±5.12	15.09±5.07	17.15±3.58	15.34±5.04	14.29±4.51	19.42±7.15
Cognition	14.64±5.70*	15.51±5.80*	—	14.97±5.85*	14.53±5.56*	13.14±2.81*	14.49±5.41*	15.20±6.09*	16.80±6.64*
Excitement/hostility	12.41±4.80*	13.16±4.39*	—	12.73±4.78	12.13±4.82	12.35±2.50	12.12±4.84	13.44±4.27	13.43±5.34

***** p<0.05, compared with each genotype, LSD tests.
